# Environmental pollutant awareness, food handling practices, and risk communication among market stakeholders in Kwara State, Nigeria: a cross-sectional survey

**DOI:** 10.3389/fpubh.2026.1922269

**Published:** 2026-09-23

**Authors:** G. K. Olawepo, A. T. Mahmud, F. O. Muhammadjamiu, S. A. Adeyemi, N. O. Lawal, S. O. Sa'ad

**Affiliations:** Department of Plant Biology, University of Ilorin, Ilorin, Nigeria

**Keywords:** environmental pollutants, food safety, market stakeholders, public health, risk communication

## Abstract

**Introduction:**

Environmental pollutants threaten food safety and public health, particularly within market environments where food handling and distribution occur. This study examined environmental pollutant awareness among market stakeholders and its implications for public health risk communication in selected markets in Kwara State, Nigeria.

**Methods:**

A cross-sectional survey was conducted among 120 respondents selected from Ganmo, Oke-Oyi, and Oko-Olowo markets using structured questionnaires. Data were analyzed using descriptive statistics and chi-square tests at a 5% significance level.

**Results:**

The respondents were predominantly female (66.7%), married (78.3%), and vendors (69.2%). Environmental pollutant awareness was high across the three markets, with all respondents reporting awareness of associated risks. Risk perception varied by location, with strong agreement regarding environmental risks reaching 100.0% in Oke-Oyi. Media channels constituted the major communication source across the markets (60.0–67.5%). Location (χ^2^ = 9.202, *p*= 0.010) and risk perception (χ^2^ = 66.930, *p* < 0.001) were significantly associated with environmental pollutant awareness.

**Conclusion:**

Environmental pollutant awareness was universally reported among market stakeholders, whereas risk perception and food-handling practices varied across markets and respondent characteristics. The observed associations between respondents' characteristics and risk perception should be interpreted as unadjusted associations rather than causal effects. Strengthening location-specific risk communication, media-based environmental education, and market-focused food safety interventions is recommended to support improved risk understanding and safer food-handling practices.

## Introduction

Many environmental food pollutants originate from substances that provide essential agricultural, industrial, and societal benefits (1). However, improper use, environmental release, and poor management transform pesticides, petroleum products, plastics, heavy metals, and microorganisms into food contaminants (2). Through air, soil, water, and food-handling pathways, these pollutants compromise food safety and pose significant public health risks (3), thereby shifting research attention from contaminant detection to food safety awareness and effective public health risk communication.

At the global level, studies have demonstrated that awareness and perceptions of environmental health risks are important components of environmental-health communication and informed decision-making. Shin et al. (4), studying U.S. adults, Ramírez et al. (5), investigating general community members and vulnerable populations in California, USA, and De Castro et al. (6), examining residents of contaminated sites and institutional stakeholders in Italy, reported that environmental awareness, risk perception, and stakeholder engagement are important considerations in environmental health communication. However, these studies were conducted outside market settings and predominantly within developed-country contexts, leaving limited evidence on environmental pollutant awareness and risk perception among market stakeholders in developing-country settings.

Similarly, studies conducted in Nigeria have examined environmental awareness and risk communication among different population groups. Isangadighi and Ukudo (7), in Agbarho, Delta State, and Adekola et al. (8), in oil-producing communities of the Niger Delta, reported inadequate environmental awareness and ineffective risk communication. Likewise, Omoyajowo et al. (9), studying rice market stakeholders across four Nigerian communities, and Madu et al. (10), investigating chemical market operators, reported low awareness of environmental pollutants and associated health risks. In Bodija Market, Ibadan, Taiwo and Ajayi (11) further observed that environmental awareness did not necessarily translate into appropriate waste-management practices. Although these studies provide valuable evidence, they have focused on specific environmental hazards, community populations, or particular market groups, leaving limited evidence on the broader awareness and risk perception of environmental pollutants among market stakeholders.

In Kwara State, existing studies similarly indicate important gaps in environmental awareness and risk communication. Olawepo et al. (12) reported low awareness of health risks associated with pesticide residues among market consumers in Ilorin, while Osagbemi et al. (13) found limited knowledge of the health hazards associated with indoor air pollution among rural residents in the state. Similarly, Emmanuel et al. (14) identified deficiencies in environmental communication capacity among environmental health students, whereas Adebayo (15) reported that market women relied predominantly on informal sources of health information. These studies suggest that environmental awareness and risk communication remain important concerns in Kwara State. However, previous research has largely examined individual environmental hazards or specific population groups, with comparatively limited attention to environmental pollutant awareness and risk perception among market stakeholders across different market locations.

Furthermore, there is limited empirical evidence examining whether reported risk perception differs according to the socio-demographic and contextual characteristics of market stakeholders. Understanding these observed patterns is important because market stakeholders operate within distinct occupational, environmental, and information contexts that may shape how environmental risks are perceived and communicated. Evidence on self-reported food-handling practices and sources of environmental-health information can provide additional contextual information for interpreting these findings and informing risk-communication strategies.

Therefore, the primary objective of this study was to assess reported awareness of environmental pollutants among market stakeholders in selected markets in Kwara State, Nigeria. Secondary objectives were to describe respondents' environmental pollutant risk perceptions, self-reported food-handling practices, and sources and preferences for public health risk communication, and to examine observed associations between risk perception and selected socio-demographic and contextual characteristics. The study did not measure environmental pollutant concentrations, contamination burdens, or objective food-safety outcomes. Accordingly, the findings are interpreted as survey-based evidence of reported awareness, perceptions, practices, and communication patterns rather than direct evidence of environmental contamination or causal effects. Environmental pollutant awareness was assessed using individual dichotomous Yes/No items and, because all respondents reported awareness, was summarized descriptively rather than subjected to inferential modeling. Risk perception, which showed variation among respondents, was therefore examined as the principal inferential outcome in relation to selected respondent characteristics.

## Materials and methods

### Study area

The study was conducted in three markets located within three local government areas in Ilorin, the capital city of Kwara State in North-Central Nigeria, namely Ganmo, Oke-Oyi, and Oko-Olowo markets. Ilorin is situated at latitude 8°30′N and longitude 4°33′E and covers an estimated land area of approximately 100 km^2^. Although officially classified within the Guinea Savannah ecological zone, the city experiences a tropical climate characterized by distinct wet and dry seasons. Annual rainfall averages about 1,200 mm, with peak rainfall occurring in August, while the harmattan season, characterized by dry and dusty northeasterly winds, extends from November to March (16). Spatial mapping and geographic analyses of the study area were generated using ArcGIS Pro (Version 3.1, Esri Inc., Redlands, California, United States) based on the World Geodetic System 1984 (WGS 84 / UTM Zone 31N) coordinate reference system, utilizing administrative boundary data sourced from the Office of the Surveyor-General, Kwara State and the National Boundaries Commission, Nigeria, alongside historical satellite imagery from Google Earth Pro accessed in January 2026, with market locations represented as approximate spatial centroids of the respective commercial markets rather than exact micro-polygon boundaries or GPS point intercepts (Figure 1).

**Figure 1:**
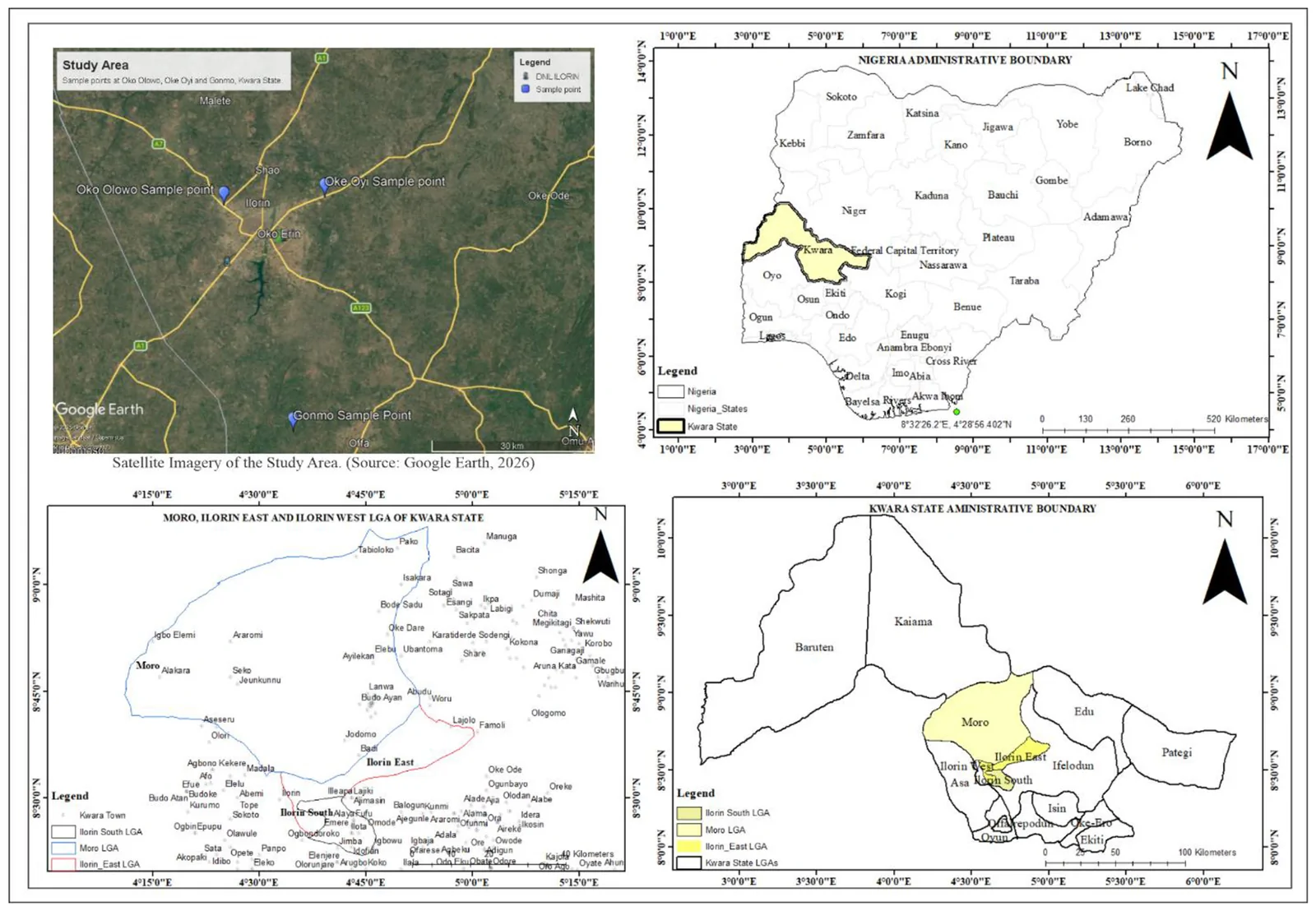
GIS Map of Ilorin, Kwara State, showing the location of the study area and the selected markets.

The selection of these specific study sites was purposive, based on the following predetermined criteria:

- Commercial Scale and Significance: They represent major commercial hubs functioning as primary distribution and retail centers for agricultural produce, food items, and household commodities within their respective local government areas in Ilorin and its periphery.
- Geographic and Locational Diversity: The selection deliberately encompasses distinct peri-urban and urban-fringe contexts, allowing for a comprehensive cross-section of market stakeholder interactions across different environmental and infrastructural settings.
- Operational Accessibility: High daily foot traffic and active stakeholder engagement (traders, vendors, transporters, and consumers) ensured sufficient data density and feasibility for the survey protocol.

Administratively, while all three markets serve the core Ilorin economic and consumer hub, they are distributed across distinct local government areas within the metropolitan and suburban fringe:

- Oke-Oyi Market is located within Ilorin East Local Government Area.
- Oko-Olowo Market is situated within Ilorin West Local Government Area.
- Ganmo Market is located within Ifelodun Local Government Area (forming part of the immediate southeastern peri-urban fringe of the Ilorin metropolis).

Furthermore, the classification criteria for location types across the survey are defined as follows:

- Urban: High population density, fully built-up commercial infrastructure, centralized public utilities, and continuous commercial activity.
- Peri-urban: Transitional zones at the edge of the urban core characterized by mixed land use (agrarian and commercial), moderate population density, and developing infrastructure.
- Rural: Low population density, agrarian-dominated economic activities, dispersed settlements, and limited urban infrastructure.

### Study design

This study employed a cross-sectional survey design to assess reported awareness, risk perceptions, practices, and public health risk communication patterns among market stakeholders. The reporting of this observational study adheres strictly to the STROBE guidelines for cross-sectional studies.

### Participant eligibility and exclusion criteria

- **Inclusion criteria:** Consenting adult market stakeholders aged 18 years and above (minimum age requirement) actively operating within the selected markets (including traders, vendors, food handlers, transporters, and consumers) for a minimum duration of 6 months.
- **Exclusion criteria:** Individuals below 18 years of age, non-consenting individuals, transient visitors, and casual passersby not involved in regular commercial or market transactions.
- **Recruitment outcomes and response rate**: Field recruitment over the 2-week period yielded a total of 135 individuals approached. Of these, 15 individuals declined participation primarily due to time constraints during active trading hours.
- **Incomplete questionnaires excluded:** Following daily field audits, 0 incomplete questionnaires were accepted; any submissions containing major missing variables or unresolvable contradictions were screened out prior to entry.
- **Final response rate:** This recruitment process yielded 120 completed and valid questionnaires, representing a final response rate of 88.9% (40 valid respondents per market stratum).

### Sample size rationale and recruitment

To ensure adequate representation across the study area, a sample-size target of 120 respondents was established, utilizing a stratified proportionate approach comprising 40 respondents from each of the three market strata.

Field recruitment was conducted over a 2-week data-collection period from June 1st to June 14th, 2026. A systematic intercept protocol was implemented to construct the sampling frame by approaching every fifth consenting adult stakeholder across designated active trading corridors within each market. A total of 135 eligible stakeholders were formally approached, of whom 15 declined participation primarily due to time constraints during active trading hours. This yielded 120 completed and valid questionnaires, representing a final response rate of 88.9% (40 valid respondents per market stratum). The sample size of 120 respondents (40 per market stratum) was determined using Cochran's formula for categorical data in cross-sectional surveys, assuming a 50% response distribution for maximum variability, a 95% confidence level, and a 9% margin of error, while accounting for logistical constraints and feasibility across the active trading environments.

### Sample and sampling technique

To ensure adequate comparative representation across the study area, the study employed a stratified sampling technique with equal allocation across three selected markets: Ganmo, Oke-Oyi, and Oko-Olowo. Each market constituted an independent sampling stratum (yielding 40 respondents per market, for a total sample size of 120 respondents). Because official, complete census frames of the total daily active population for each market are dynamic and unrecorded, equal allocation across strata was selected over proportionate allocation to ensure sufficient statistical power and robust comparative analysis across all market locations.

To operationalize this in the field, a systematic intercept approach was utilized to construct an on-site sampling frame by intercepting every fifth consenting adult stakeholder across active trading zones within each stratum. From this frame, simple random sampling (via a randomized lottery draw) was applied to select eligible participants, providing equal opportunities for participation and mitigating selection bias.

### Data collection and administration protocol

Data for this study were collected using a newly developed structured questionnaire designed specifically to address the research objectives. The instrument was structured into five distinct sections (A–E) covering respondents' socio-demographic characteristics, awareness of environmental pollutants, risk perceptions, food safety and handling practices, and public health risk communication preferences. The complete questionnaire has been provided as Supplementary material to ensure full transparency and replicability.

Data were collected using a structured, pre-tested questionnaire administered by trained research assistants fluent in both English and Yoruba (the primary local language). To accommodate participants with limited formal literacy and minimize information bias, items and Likert-scale options were translated, read, and explained verbally in Yoruba or Nigerian Pidgin using standardized, non-leading protocols designed to preserve conceptual equivalence.

### The instrument comprised five sections

1. Section A (Socio-Demographic Characteristics): Captured gender, age, educational level, marital status, market role, residential location, household characteristics, and monthly income level.
2. Section B (Environmental Pollutant Awareness): Assessed reported awareness of multi-faceted environmental pollutants and food contamination pathways, including heavy metals, soot and smoke, microplastics, pesticide residues, and microbial contaminants. Awareness was assessed using dichotomous Yes/No items covering heavy metals, soot and smoke, microplastics, pesticide residues, and microbial contaminants.
3. Section C (Risk Perception): Measured risk perceptions regarding environmental pollutants across dimensions of perceived severity, vulnerability, health consequences, susceptibility, and socioeconomic impacts using a 5-point Likert scale (1 = Strongly Disagree to 5 = Strongly Agree).
4. Section D (Food Safety Practices): Examined food protection methods, contamination avoidance, decontamination practices, and safety priorities via multi-response and rating items.
5. Section E (Public Health Risk Communication): Evaluated information sources, channel trust, preferred communication formats, accessibility, language preferences, and perceived effectiveness of existing communication campaigns.

### Handling of missing data and quality control

Field-level quality control was enforced through daily audits of all retrieved questionnaires. Submissions containing major missing variables, unresolvable contradictions, or incomplete core sections were systematically excluded prior to data entry. Consequently, the final analyzed dataset of 120 respondents contained complete data for all evaluated variables, precluding the need for imputation techniques.

### Validation, reliability, and bias control

Face and content validity of the research instrument were established through expert review by specialists in environmental health, public health, and research methodology. The experts reviewed the questionnaire for clarity, relevance, appropriateness, comprehensiveness, and alignment of the items with the study objectives and intended domains. Based on the expert assessment, items were reviewed and refined to ensure that the questionnaire adequately represented the areas of environmental pollutant awareness, risk perception, food-handling practices, and public health risk communication.

Instrument reliability was evaluated through a pilot study involving 20 market stakeholders from an auxiliary market that was not included in the main survey. Because the questionnaire comprised multiple domains and included both scale-based and categorical items, internal consistency reliability was assessed separately for applicable multi-item scales rather than by calculating a single overall reliability coefficient. Single-item demographic questions, categorical variables, and check-all-that-apply multiple-response items were excluded from Cronbach's alpha calculations. Cronbach's alpha coefficients were calculated for the applicable multi-item domains as follows: Section B (Environmental Pollutant Awareness, 8 items): α = 0.76; Section C (Risk Perception, 10 items): α = 0.82; Section D (Food Safety Practices, 6 rating items): α = 0.75; and Section E (Public Health Risk Communication, 7 items): α = 0.79. All evaluated multi-item scales exceeded the predetermined acceptable threshold, indicating satisfactory internal consistency.

#### Construct operationalization

The constructs measured in the questionnaire were operationalized a priori according to the study objectives and the conceptual domains represented by the questionnaire sections. Environmental pollutant awareness was assessed through items addressing awareness of environmental contamination and specific pollutants. Risk perception was assessed using Likert-type statements addressing perceived severity, susceptibility or vulnerability, and socio-economic consequences of environmental pollutants. Food-handling practices were assessed through respondents' reported methods of protecting, preparing, decontaminating, and transporting food, with relevant responses subsequently categorized according to the operational definitions described in the Data Analysis and Statistical Methods section. Public health risk communication was assessed through information sources, trusted information providers, preferred communication formats, communication frequency, preferred language, and perceived effectiveness of existing communication.

Formal construct validity was not evaluated using exploratory factor analysis, confirmatory factor analysis, or another statistical construct-validation procedure. Therefore, the study does not claim empirical validation of the underlying factor structure or dimensionality of the multi-item domains. Instead, evidence supporting the adequacy of the constructs was based on their a priori operationalization, alignment of questionnaire items with the study objectives, and expert assessment of item relevance and coverage. This distinction clarifies that the study established face and content validity and assessed internal consistency reliability, but did not formally establish empirical construct validity.

To minimize potential recall and social desirability biases, research assistants explained the purpose of the study, assured participants of the confidentiality of their responses, and maintained neutrality during questionnaire administration. The absence of formal construct-validation procedures is acknowledged as a limitation of the study.

### Primary and secondary outcomes

The prespecified primary outcome was environmental pollutant awareness, assessed using eight dichotomous Yes/No items covering awareness of heavy metals, soot/smoke, microplastics, pesticide residues, and microbial contaminants. Each item was coded as 1 for “Yes” (aware) and 0 for “No” (not aware) and analyzed individually using frequencies and percentages. No composite awareness score or graded awareness category was used.

Secondary outcomes included risk perception, food-handling practices, and public health risk communication. Risk perception was assessed using the 10-item risk-perception section, food-handling practices were classified as poor, fair, good, or excellent according to the operational coding described above, and public health risk communication was assessed using the seven-item communication section.

### Data analysis and statistical methods

Data were coded and analyzed using the Statistical Package for the Social Sciences (SPSS) version 25.0, with Microsoft Excel 2016 utilized for visualization. Descriptive statistics (frequencies and percentages) were used to summarize sociodemographic characteristics, reported environmental pollutant awareness, risk perception, food-handling practices, and communication channels. Responses to the environmental pollutant awareness items were summarized descriptively. Because all respondents answered “Yes” to all awareness items, resulting in 100% reported awareness across the assessed pollutant categories, no comparative or inferential analysis was performed for the awareness outcome. Pearson's chi-square tests were subsequently used to examine associations between selected respondent characteristics and environmental pollutant risk perception, with Cramér's V used to quantify effect sizes.

#### Income

Average monthly income was assessed using four predefined categories: below ₦50,000, ₦50,000–₦100,000, ₦101,000–₦250,000, and above ₦250,000. The cut-offs were selected with reference to poverty-threshold considerations and to provide meaningful socioeconomic groupings for the study population. The use of categorical income bands also reduced the need for respondents to disclose an exact income amount and facilitated comparison across socioeconomic groups. Income was treated as an ordered categorical variable in the analysis. The questionnaire recorded “average monthly income” without specifying whether the reported amount was gross or net or whether it represented personal, household, or business income; these distinctions were therefore not imposed during analysis.

#### Assessment of environmental pollutant awareness (Section B)

Environmental pollutant awareness was assessed using eight dichotomous Yes/No items covering heavy metals, soot/smoke, microplastics, pesticide residues, and microbial contaminants. For each item, “Yes” was coded as 1, indicating reported awareness, while “No” was coded as 0, indicating no reported awareness. Responses were initially assessed at the item level using frequencies and percentages. Because all respondents (100%) answered “Yes” to all eight awareness items, indicating no variation across the assessed pollutant categories, the awareness findings were summarized in the Results as universal reported awareness rather than presented as separate item-level comparisons.

#### Assessment and categorization of food-handling practices (Section D)

Food-handling practices were assessed using six response options representing the methods used by respondents to prepare or decontaminate food. The responses were coded as follows: 1 = washing with plain water, 2 = washing with additives, 3 = parboiling, 4 = peeling, 5 = brushing, and 6 = none. For analysis, the responses were classified according to the operational practice categories used in the study: parboiling was classified as excellent practice; washing with plain water and washing with additives were classified as good practice; peeling and brushing were classified as fair practice; and reporting no food-handling practice was classified as poor practice. The resulting categories were summarized using frequencies and percentages, and their distributions across the three markets were examined using cross-tabulations.

#### Operationalization and categorization of variables presented in the figures

Risk-perception items were initially assessed using a five-point Likert scale: 1 = strongly disagree, 2 = disagree, 3 = neutral, 4 = agree, and 5 = strongly agree. For the chi-square analyses, risk perception was treated as a categorical dependent variable and the observed responses were represented in three categories: Neutral, Agree, and Strongly Agree. In the analyzed dataset, these categories comprised 12 (10.0%), 19 (15.8%), and 89 (74.2%) respondents, respectively. Thus, “strong agreement” refers specifically to respondents who selected “strongly agree” (score 5). Information and media sources were assessed using seven predefined multiple-response options. For analysis, these were grouped into three derived categories: media channels, comprising traditional media and digital media; formal channels, comprising religious institutions, market/trade union meetings, community town criers or megaphones, and government health officers/sanitarians; and interpersonal channels, comprising family, friends, and neighbors. Other communication variables, including preferred communication format, communication frequency, and preferred language, were retained according to their predefined questionnaire response categories.

#### Inferential analysis

Pearson's chi-square tests of independence were used to examine associations between respondents' characteristics and categorical study variables. For the specific analysis of environmental pollutant risk perception, risk perception was entered as the dependent categorical variable, while gender, age group, education, marital status, parenting status, income, market role, and location were entered as independent categorical variables. Expected cell frequencies were examined to assess the assumptions of the chi-square test before interpreting the results. Pearson's χ^2^ statistic, degrees of freedom, and corresponding *p-values* were reported. Cramér's V was calculated as a measure of effect size and reported with its 95% confidence interval.

#### Exploratory nature of multiple associations

Because multiple categorical associations were examined, the chi-square analyses were considered exploratory rather than confirmatory. The analyses were undertaken to identify potential patterns of association between respondents' characteristics and the study variables. The number of hypothesis tests was recognized as increasing the potential for type I error; therefore, the findings were interpreted cautiously, with emphasis placed primarily on the magnitude and precision of associations, as reflected by Cramér's V and its 95% confidence interval, rather than on statistical significance alone.

#### Confidence interval estimation

To communicate the precision of the estimates, 95% confidence intervals (CIs) were calculated for relevant proportions reported in the Results using the Wilson method. For categorical association analyses, 95% confidence intervals for Cramér's V were estimated using 2,000 bootstrap replications. Statistical significance was assessed at the 5% level, while 95% CIs were used to indicate the precision of the estimates. Detailed contingency tables showing the observed frequencies for the environmental pollutant risk-perception analyses are provided in Supplementary Tables S1–S8 to facilitate independent assessment of the chi-square results.

## Results

### Demographic and socioeconomic characteristics of respondents across study locations

The demographic and socioeconomic characteristics of the 120 respondents across Oke-Oyi, Oko-Olowo, and Ganmo are presented in Table 1. Females constituted the majority of respondents (66.7%), although gender representation was balanced in Ganmo. The 26–40 age group was the most represented (51.7%), while 78.3% of respondents were married. Vendors constituted the largest market-role group (69.2%). Educational attainment varied, with primary education being most common (30.8%), while 22.5% had no formal education. Most respondents were from urban areas (59.2%) and were parents (75.8%). Half of the respondents (50.8%) reported a monthly income below ₦50,000.

**Table 1:** Demographic and socioeconomic characteristics of respondents across study locations.

Variables	Oke-Oyi	Oko-Olowo	Ganmo	Total, *n* (%) [95% CI]
Gender				
Male	6 (15.0%)	14 (35.0%)	20 (50.0%)	40 (33.3%) [25.5–42.2]
Female	34 (85.0%)	26 (65.0%)	20 (50.0%)	80 (66.7%) [57.8–74.5]
Age group				
18–25	6 (15.0%)	6 (15.0%)	7 (17.5%)	19 (15.8%) [10.4–23.4]
26–40	20 (50.0%)	20 (50.0%)	22 (55.0%)	62 (51.7%) [42.8–60.4]
41–60	12 (30.0%)	12 (30.0%)	9 (22.5%)	33 (27.5%) [20.3–36.1]
60+	2 (5.0%)	2 (5.0%)	2 (5.0%)	6 (5.0%) [2.3–10.5]
Education				
None	6 (15.0%)	11 (27.5%)	10 (25.0%)	27 (22.5%) [15.9–30.8]
Primary	16 (40.0%)	13 (32.5%)	8 (20.0%)	37 (30.8%) [23.3–39.6]
Secondary	14 (35.0%)	10 (25.0%)	9 (22.5%)	33 (27.5%) [20.3–36.1]
Tertiary	4 (10.0%)	6 (15.0%)	13 (32.5%)	23 (19.2%) [13.1–27.1]
Market role				
Consumer	13 (32.5%)	6 (15.0%)	6 (15.0%)	25 (20.8%) [14.5–28.9]
Vendor	25 (62.5%)	32 (80.0%)	26 (65.0%)	83 (69.2%) [60.4–76.7]
Transporter	2 (5.0%)	2 (5.0%)	2 (5.0%)	6 (5.0%) [2.3–10.5]
Official	0 (0.0%)	0 (0.0%)	6 (15.0%)	6 (5.0%) [2.3–10.5]
Location type				
Urban	8 (20.0%)	40 (100.0%)	23 (57.5%)	71 (59.2%) [50.2–67.5]
Peri-urban	10 (25.0%)	0 (0.0%)	9 (22.5%)	19 (15.8%) [10.4–23.4]
Rural	22 (55.0%)	0 (0.0%)	8 (20.0%)	30 (25.0%) [18.1–33.4]
Marital status				
Single	5 (12.5%)	6 (15.0%)	11 (27.5%)	22 (18.3%) [12.4–26.2]
Married	35 (87.5%)	32 (80.0%)	27 (67.5%)	94 (78.3%) [70.1–84.8]
Divorced/Widowed	0 (0.0%)	2 (5.0%)	2 (5.0%)	4 (3.3%) [1.3–8.3]
Parenting status				
Parent	24 (60.0%)	36 (90.0%)	31 (77.5%)	91 (75.8%) [67.4–82.6]
Non-parent	16 (40.0%)	4 (10.0%)	9 (22.5%)	29 (24.2%) [17.4–32.6]
Income				
< ₦50,000	22 (55.0%)	22 (55.0%)	17 (42.5%)	61 (50.8%) [42.0–59.6]
₦50,000–₦100,000	13 (32.5%)	6 (15.0%)	7 (17.5%)	26 (21.7%) [15.2–29.9]
₦101,000–₦250,000	3 (7.5%)	6 (15.0%)	8 (20.0%)	17 (14.2%) [9.0–21.5]
>₦250,000	2 (5.0%)	6 (15.0%)	8 (20.0%)	16 (13.3%) [8.4–20.6]

### Environmental pollutant risk awareness among respondents across the three study markets

Reported awareness of environmental pollutants was universal among the 120 respondents (100%). All respondents indicated awareness of each of the environmental pollutant categories assessed, including heavy metals, soot and smoke, microplastics, pesticide residues, and microbial contaminants. Because all awareness items received a “Yes” response from every respondent, no meaningful variation existed at the item level; consequently, the awareness findings were summarized rather than presented as a separate figure.

### Distribution of risk perception across the three study locations

Figure 2 presents the distribution of respondents' risk perception across the three study locations, while Table 2 provides the corresponding frequencies, percentages, and 95% confidence intervals. Strong agreement was most prevalent in Oke-Oyi (100.0%), followed by Ganmo (75.0%) and Oko-Olowo (47.5%). In contrast, neutral perception was most prevalent in Oko-Olowo (30.0%) and absent in Ganmo and Oke-Oyi, while agreement was highest in Ganmo (25.0%) and absent in Oke-Oyi.

**Figure 2:**
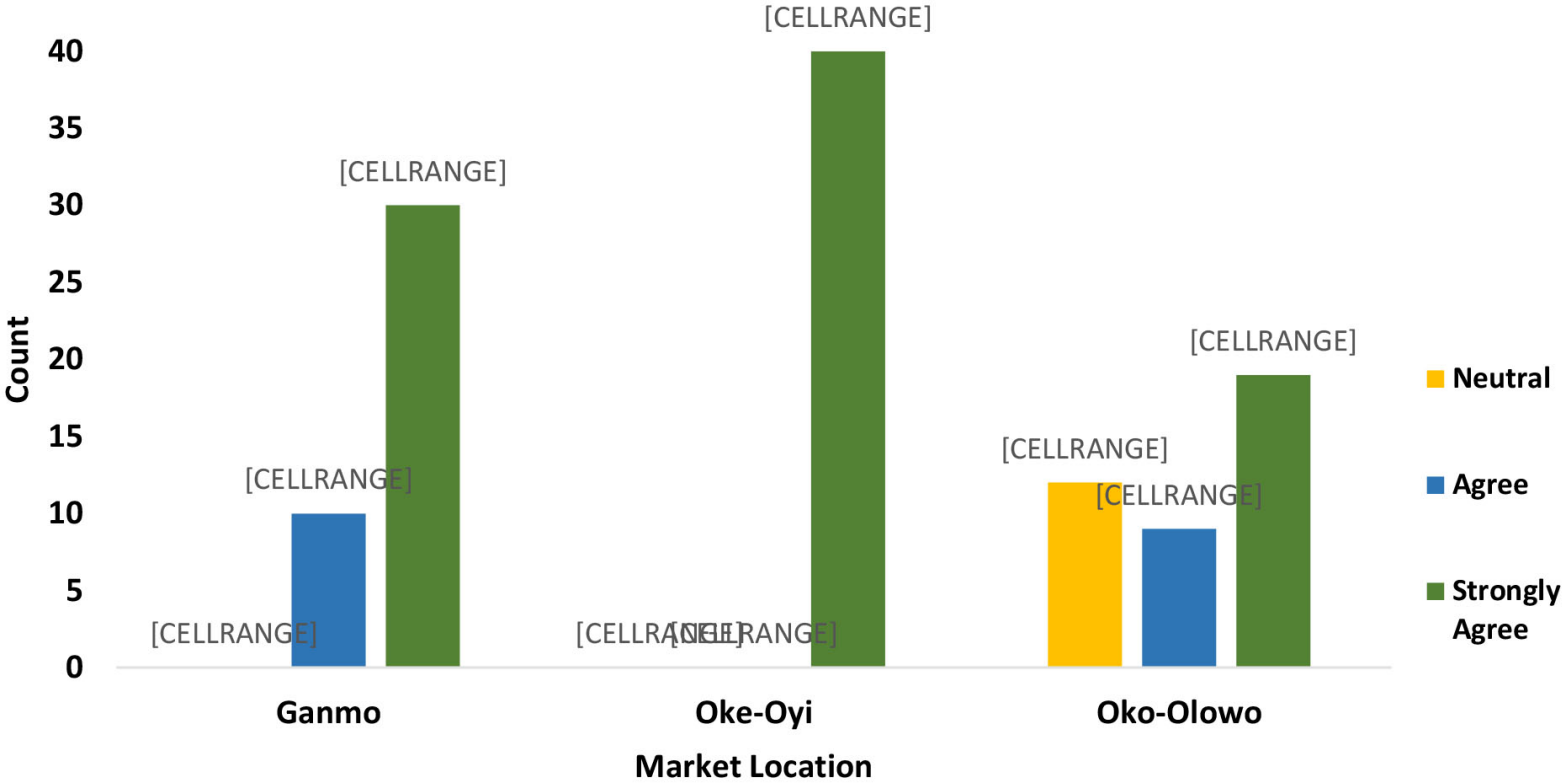
Distribution of risk perception across the three study locations.

**Table 2:** Distribution of risk perception categories by market with 95% confidence intervals.

Market	Neutral, *n* (%)	95% CI	Agree, *n* (%)	95% CI	Strongly agree, *n* (%)	95% CI
Ganmo	0 (0.0%)	0.0%−8.8%	10 (25.0%)	14.2%−40.2%	30 (75.0%)	59.8%−85.8%
Oke-Oyi	0 (0.0%)	0.0%−8.8%	0 (0.0%)	0.0%−8.8%	40 (100.0%)	91.2%−100.0%
Oko-Olowo	12 (30.0%)	18.1%−45.4%	9 (22.5%)	12.3%−37.5%	19 (47.5%)	32.9%−62.5%

Values are presented as n (%). CI, confidence interval. Percentages are based on 40 respondents per market. 95% CIs were calculated using the Wilson method.

### Comparative analysis of food handling practices across three markets

Figure 3 presents a comparative analysis of food-handling practices across the three study markets, while Table 3 provides the corresponding frequencies, percentages, and 95% confidence intervals. Poor handling practices were most prevalent in Ganmo (47.5%), whereas good practices were most prevalent in Oke-Oyi (45.0%). Oko-Olowo recorded the highest proportions of fair and excellent practices (25.0% each) but the lowest proportion of good practices (12.5%). Excellent practices were least prevalent in Ganmo (5.0%).

**Figure 3:**
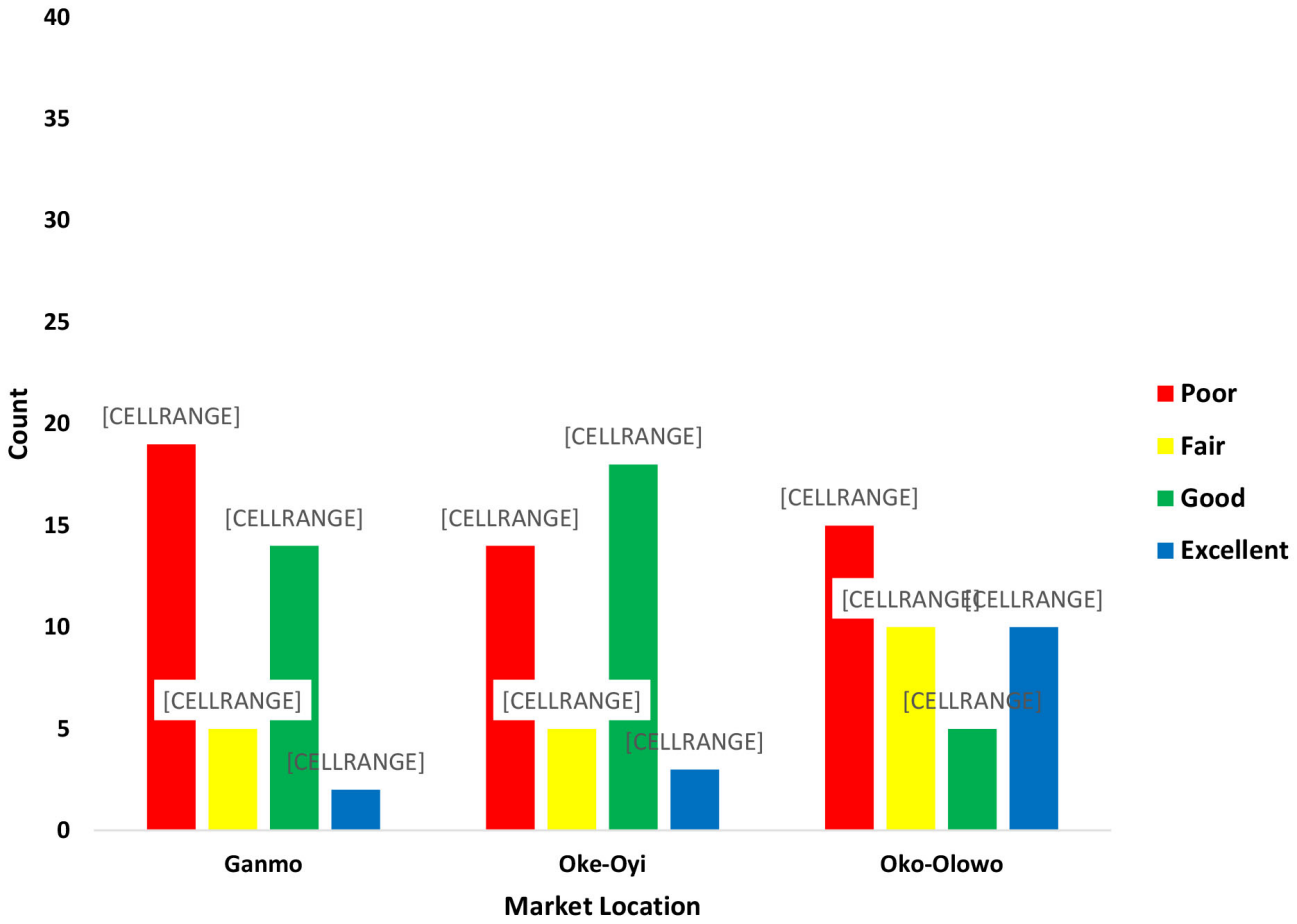
Comparative analysis of food handling practices across the three surveyed markets.

**Table 3:** Distribution of food-handling practice ratings across study markets with 95% confidence intervals.

Market	Poor, *n* (%)	95% CI	Fair, *n* (%)	95% CI	Good, *n* (%)	95% CI	Excellent, *n* (%)	95% CI
Ganmo	19 (47.5%)	32.9%−62.5%	5 (12.5%)	5.5%−26.1%	14 (35.0%)	22.1%−50.5%	2 (5.0%)	1.4%−16.5%
Oke-Oyi	14 (35.0%)	22.1%−50.5%	5 (12.5%)	5.5%−26.1%	18 (45.0%)	30.7%−60.2%	3 (7.5%)	2.6%−19.9%
Oko-Olowo	15 (37.5%)	24.2%−53.0%	10 (25.0%)	14.2%−40.2%	5 (12.5%)	5.5%−26.1%	10 (25.0%)	14.2%−40.2%

Values are presented as n (%). CI, confidence interval. Percentages are based on 40 respondents per market. 95% CIs were calculated using the Wilson method.

### Distribution of preferred environmental pollutant risk communication channels across three markets

Figure 4 presents a comparative analysis of food-handling practices across the three study markets, while Table 4 provides the corresponding frequencies, percentages, and 95% confidence intervals. Poor practices were most prevalent in Ganmo (47.5%), whereas Oke-Oyi had the highest proportion of good practices (45.0%). Oko-Olowo recorded the highest proportions of fair (25.0%) and excellent (25.0%) practices, but also had the lowest proportion of good practices (12.5%). Excellent practices were least prevalent in Ganmo (5.0%).

**Figure 4:**
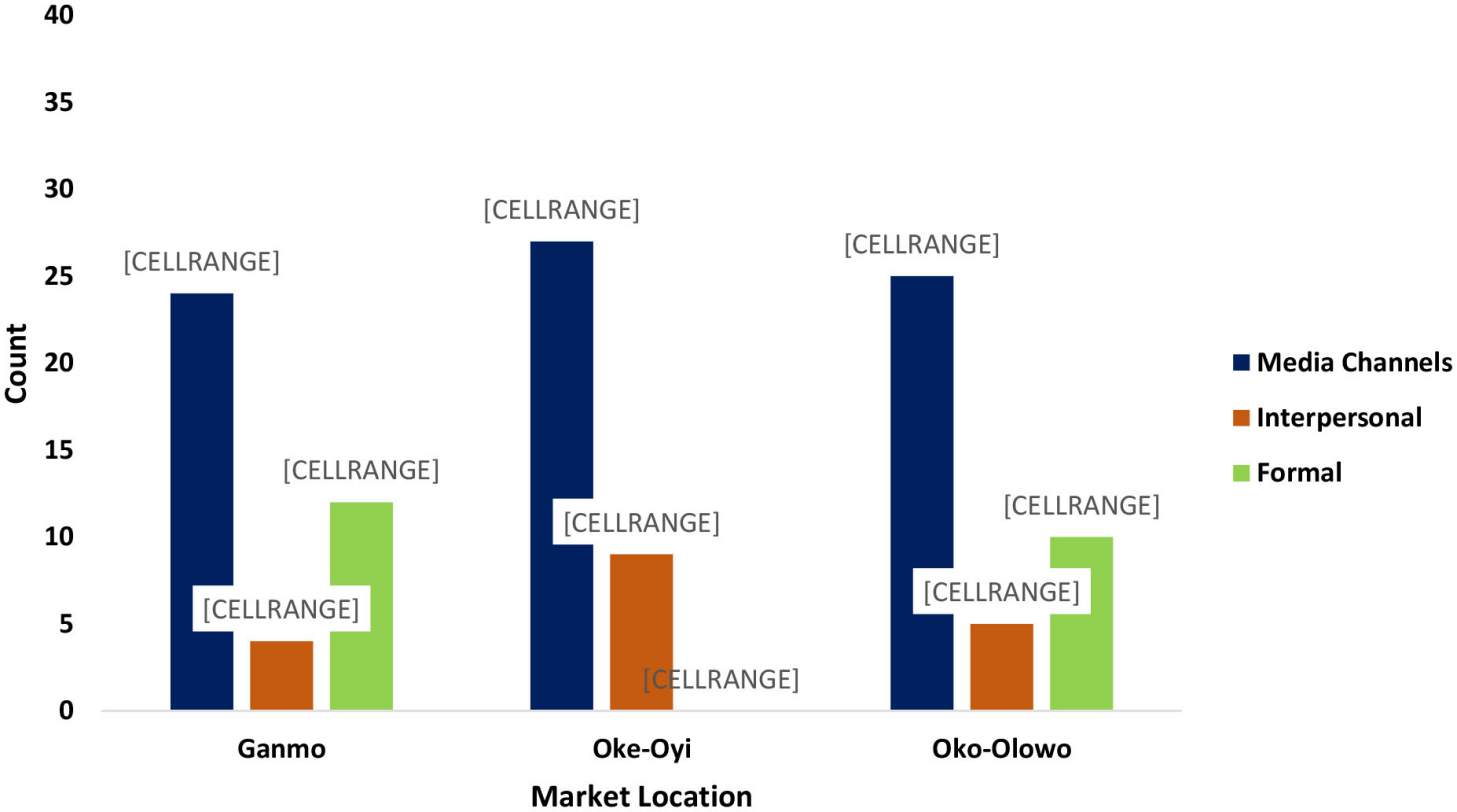
Distribution of preferred environmental pollutant risk communication channels across three markets.

**Table 4:** Distribution of preferred environmental pollutant risk communication channels across study markets with 95% confidence intervals.

Market	Media, *n* (%)	95% CI	Interpersonal, *n* (%)	95% CI	Formal, *n* (%)	95% CI
Ganmo	24 (60.0%)	44.6%−73.8%	4 (10.0%)	4.0%−22.6%	12 (30.0%)	18.1%−45.4%
Oke-Oyi	27 (67.5%)	52.0%−79.9%	9 (22.5%)	12.3%−37.5%	0 (0.0%)	0.0%−8.8%
Oko-Olowo	25 (62.5%)	47.1%−75.8%	5 (12.5%)	5.5%−26.1%	10 (25.0%)	14.2%−40.2%

Values are presented as n (%). CI, confidence interval. Percentages are based on 40 respondents per market. 95% CIs were calculated using the Wilson method.

### Association of some variables with environmental pollutant risk perception

Table 5 presents the associations between respondents' characteristics and environmental pollutant risk perception. All examined characteristics were significantly associated with risk perception (*p* < 0.05). Gender showed the strongest association (Cramér's *V* = 0.648), followed by marital status (*V* = 0.468), location (*V* = 0.413), and age group (*V* = 0.389). Parenting status, income, market role, and education showed comparatively weaker associations, with Cramér's V values ranging from 0.297 to 0.333.

**Table 5:** Chi-square associations, effect sizes (Cramér's V), and 95% confidence intervals for factors associated with environmental pollutant risk perception.

Variable	*χ2*	*df*	*p*-value	Cramér's V	95% CI (lower, upper)
Gender	50.331	2	< 0.001	0.648	[0.514, 0.781]
Marital status	52.572	4	< 0.001	0.468	[0.353, 0.578]
Location	41.017	4	< 0.001	0.413	[0.335, 0.503]
Age group	36.245	6	< 0.001	0.389	[0.299, 0.514]
Parenting status	13.320	2	< 0.001	0.333	[0.256, 0.412]
Income	25.935	6	< 0.001	0.329	[0.265, 0.412]
Market role	25.828	6	< 0.001	0.328	[0.216, 0.460]
Education	21.171	4	< 0.001	0.297	[0.217, 0.403]

Environmental pollutant risk perception was the dependent categorical variable and comprised three response categories: Neutral (*n* = 12, 10.0%), Agree (*n* = 19, 15.8%), and Strongly Agree (*n* = 89, 74.2%). χ^2^, Pearson chi-square statistic; df, degrees of freedom; CI, confidence interval for Cramér's V. The 95% confidence intervals for Cramér's V were estimated using 2,000 bootstrap replications. Statistical significance was accepted at *p* < 0.05. Detailed contingency tables showing observed frequencies are provided in Supplementary Tables S1–S8.

## Discussion

The present study identified three principal findings. First, reported awareness of environmental pollutants was universal among the 120 respondents, with all participants indicating awareness of the pollutant categories assessed. Second, despite this universal awareness, environmental pollutant risk perception varied across the three markets, with strong agreement reported by 100.0% of respondents in Oke-Oyi, 75.0% in Ganmo, and 47.5% in Oko-Olowo. Third, food-handling practices were heterogeneous across markets: poor practice was most prevalent in Ganmo (47.5%), good practice was most prevalent in Oke-Oyi (45.0%), while Oko-Olowo recorded the highest proportions of fair and excellent practices (25.0% each). The chi-square analyses additionally showed statistically significant unadjusted associations between risk perception and gender, marital status, location, age group, parenting status, income, market role, and education, with Cramér's V ranging from 0.297 to 0.648. These findings collectively indicate that reported awareness, risk perception, and food-handling behavior were not uniformly distributed within the study population and, importantly, that universal reported awareness did not correspond to uniformly favorable food-handling practices.

### Environmental pollutant awareness and risk perception

The finding of 100% reported awareness is notable because previous research among market populations has generally documented variation in environmental knowledge. For example, Itasanmi and Jegede (17), in a study of 403 market women in Ibadan, Nigeria, reported good knowledge in some environmental domains, including erosion and water pollution, but comparatively limited knowledge regarding soil degradation. Their findings demonstrate that environmental knowledge can vary across specific hazard domains even within a population that generally expresses environmental awareness. The universal awareness observed in the present study may therefore reflect respondents' recognition of the existence of environmental pollutants rather than comprehensive understanding of their sources, exposure pathways, health effects, or preventive measures. This distinction is important because the present awareness measure was based on self-reported responses to dichotomous items rather than an objective test of depth of knowledge.

The present finding is also broadly consistent with evidence that awareness of environmental hazards does not necessarily imply equivalent understanding of the associated health implications or corresponding behavioral responses. Chin et al. (18), for example, found relatively high public awareness of air-pollution sources among respondents in Peninsular Malaysia, yet awareness did not uniformly translate into willingness to undertake individual protective actions. The authors noted that respondents were more supportive of environmental-protection measures that did not require substantial individual effort, illustrating the distinction between recognizing an environmental problem and engaging in personally demanding protective behavior. Similarly, Ramírez et al. (5) emphasized that effective environmental-health communication requires more than simply transmitting information because environmental-health literacy involves the ability to understand, evaluate, and use health information in decision-making. Thus, the universal awareness observed in this study should not be interpreted as evidence of universal environmental-health literacy.

The difference between awareness and risk perception is particularly important. Although all respondents reported awareness, the intensity of risk perception differed markedly across the study markets. Strong agreement was highest in Oke-Oyi and lowest in Oko-Olowo. Risk perception is a multidimensional construct that may incorporate perceived severity, susceptibility, familiarity, personal experience, trust, knowledge, and perceived control over a hazard. Li and Konisky (19) demonstrated that individual characteristics can be associated with differences and misperceptions in perceptions of local environmental risks, highlighting that recognition of an environmental hazard does not necessarily result in a uniform assessment of its seriousness. Shin et al. (4) similarly emphasized the importance of understanding public perceptions of environmental public-health risks because perceptions can differ from objective environmental conditions. The present study therefore provides evidence of variation in reported risk perception, rather than evidence that respondents differed in their basic awareness of environmental pollutants.

The market-level differences should be interpreted cautiously. The higher proportion of strong agreement in Oke-Oyi and the lower proportion in Oko-Olowo indicate that the distribution of risk-perception responses differed across the sampled markets, but these differences do not establish that market location caused the observed variation. Previous Nigerian research has shown that perceptions of environmental health risks can develop within broader contexts of environmental degradation, exposure, communication, and community experience (8). Omoyajowo et al. (9) likewise examined awareness, exposure risks, and environmental attitudes relating to pesticide and heavy-metal contamination among respondents in Nigerian rice-producing communities and demonstrated that perceptions of environmental hazards are embedded within particular exposure and information contexts. These studies provide plausible contextual explanations for variation in risk perception, but exposure history, perceived severity, trust in information, and previous experiences with environmental pollution were not directly measured in the present study. They should therefore be regarded as possible explanatory factors rather than established causes of the observed market differences.

### Food-handling practices and the awareness–practice gap

Food-handling practices represent an important component of the present study because they provide a behavioral dimension that complements the universal awareness finding. In this study, the predefined practice categories classified parboiling as excellent practice; washing with plain water or additives as good practice; peeling and brushing as fair practice; and reporting no food-handling practice as poor practice. Using this classification, poor practice was most prevalent in Ganmo (47.5%), good practice was most prevalent in Oke-Oyi (45.0%), while Oko-Olowo had the highest proportions of fair and excellent practices (25.0% each). Excellent practice was least frequent in Ganmo (5.0%). The marked variation in these categories contrasts with the uniform awareness result and therefore represents an important finding in its own right.

The coexistence of universal reported awareness with substantial variation in food-handling practice suggests a potential awareness–practice gap. This pattern is consistent with the wider food-safety literature. Desye et al. (20), in a systematic review and meta-analysis of street-food vendors in low- and middle-income countries, reported pooled good knowledge of 62%, compared with approximately 51% for good food-safety practice. The difference indicates that knowledge or awareness does not necessarily translate directly into appropriate food-handling behavior. Atalay et al. (21) similarly reported, in a systematic review and meta-analysis covering food handlers in sub-Saharan Africa, that only approximately half demonstrated good food-hygiene practices. Thus, the present finding that awareness was universal while practice varied is not inconsistent with previous evidence; rather, it contributes a market-specific example of the broader separation between knowledge and behavior.

The Nigerian context provides particularly relevant comparison. Abatan et al. (22) examined hygiene and handling practices among vegetable traders in Nigeria and emphasized that food safety depends on more than the act of washing vegetables. Their cross-disciplinary analysis considered the practical and behavioral dimensions of handling and highlighted the importance of examining how traders actually handle food rather than assuming that awareness of hygiene requirements automatically guarantees safe practices. This is directly relevant to the present study because respondents were market stakeholders involved with food-related activities. The finding of substantial variation in reported practice despite universal awareness therefore supports the need to distinguish between recognizing environmental or food-safety risks and consistently implementing protective practices.

The findings of Itasanmi and Jegede (17) provide further Nigerian evidence that environmental knowledge and behavior may not be perfectly aligned. Among market women in Ibadan, the authors reported favorable environmental attitudes and responsible behavior in some domains alongside less responsible behavior in others. Their results suggest that environmental awareness should not automatically be interpreted as uniform adoption of environmentally protective behavior. This is conceptually consistent with the present study, where all respondents reported awareness but the distribution of food-handling practices ranged from poor to excellent. The present study therefore adds a food-handling dimension to evidence from Nigerian market populations showing that environmental knowledge and behavior can coexist without complete behavioral consistency.

Several mechanisms may explain why awareness does not always translate into practice. Food-handling behavior may depend on the availability of appropriate facilities and resources, perceived convenience, established routines, social expectations, confidence in the effectiveness of a particular practice, and competing practical priorities. Abatan et al. (22) is particularly relevant because it demonstrates that vegetable-handling safety involves multiple dimensions of trader behavior rather than a single hygiene action. Similarly, Desye et al. (20) and Atalay et al. (21) show at the broader regional level that adequate knowledge and favorable practice are not equivalent outcomes. These mechanisms provide scientifically plausible explanations for an awareness–practice gap, but the present questionnaire did not directly measure all of these factors. Consequently, they should not be presented as explanations established by the current data.

The market-specific differences in food-handling practice should likewise be interpreted as observed distributions rather than causal market effects. The higher prevalence of poor practice in Ganmo and the higher prevalence of good practice in Oke-Oyi may reflect differences in respondents, trading routines, access to facilities, information exposure, or other contextual conditions, but the present cross-sectional analysis cannot distinguish among these possibilities. Previous food-safety research has demonstrated substantial heterogeneity in hygiene practices across different market and vendor settings, reinforcing the need for setting-specific investigation rather than assuming a single explanation for observed differences (21, 22).

The operational categorization of food-handling practices should also be considered when interpreting these findings. The categories used in this study represent the predefined operational classification adopted for the questionnaire and should not be interpreted as a universally validated food-safety scoring system. In addition, the findings are based on self-reported practices rather than direct observation. Consequently, the results indicate differences in reported food-handling behavior and do not establish whether the practices were performed correctly, consistently, or under hygienic conditions. This limitation is particularly relevant when interpreting the awareness–practice gap because social-desirability and recall effects may influence self-reported behavioral responses.

### Associations with environmental pollutant risk perception

The chi-square analyses showed statistically significant unadjusted associations between environmental pollutant risk perception and gender, marital status, location, age group, parenting status, income, market role, and education. The effect sizes ranged from Cramér's *V* = 0.297 for education to 0.648 for gender, indicating variation in the magnitude of the observed associations. These results mean that the distribution of risk-perception responses differed across the categories of each examined characteristic within the study sample.

These associations should not, however, be interpreted as evidence that the examined characteristics caused or determined environmental pollutant risk perception. The study was cross-sectional, and the analyses were based principally on bivariate Pearson chi-square tests. No multivariable model was used to determine whether the observed associations were independent of one another or whether confounding or interaction affected the results. Cramér's V describes the strength of association between categorical variables but does not establish causality, temporal direction, or independent predictive importance. Accordingly, the appropriate interpretation is that these characteristics were significantly associated with differences in the distribution of risk-perception responses in this sample.

The presence of associations between demographic characteristics and environmental risk perception is nevertheless consistent with previous research. Li and Konisky (19) demonstrated that personal attributes can be associated with differences in perceptions and misperceptions of local environmental risks, indicating that individuals exposed to similar environmental circumstances may not necessarily evaluate risks identically. Shin et al. (4) likewise found that public perceptions of environmental public-health risks vary across populations and emphasized the importance of considering how people interpret environmental hazards rather than assuming that objective risk and perceived risk are equivalent. These findings provide a scientific basis for interpreting the present associations as plausible variations in perceived environmental risk rather than evidence of causal demographic effects.

The association observed for education is also compatible with Nigerian evidence. Itasanmi and Jegede (17), in a separate study of market women in Ibadan, reported significant differences associated with education in environmental knowledge, attitude, and behavior. However, the present study did not examine education as a determinant of risk perception, nor did it test whether the association remained after adjustment for other characteristics. Therefore, while the finding is consistent with previous Nigerian evidence that educational characteristics can be related to environmental outcomes, it should not be interpreted as evidence that education independently determines risk perception in the present sample.

The relatively high Cramér's V observed for gender requires similar caution. Previous environmental-risk research has documented variation in risk perception across gender groups, but such differences may be related to broader contextual, experiential, informational, or social factors rather than gender itself acting as a causal mechanism. Li and Konisky (19) similarly emphasized that personal attributes can be related to differences in environmental-risk perception, while Shin et al. (4) demonstrated the broader complexity of public environmental-health risk perception. Therefore, the relatively large association observed between gender and risk perception in this study should be reported as a sample-specific association, not as evidence that gender has a greater causal influence than the other characteristics.

The significant association with parenting status is also potentially relevant because family circumstances may influence how environmental health risks are perceived. However, parenting status may be correlated with age, marital status, income, or other characteristics. Since the current analysis did not adjust for these potential relationships, it is not possible to determine whether parenting status represents an independent association. The appropriate conclusion is therefore limited to the observed bivariate relationship.

### Communication, awareness, and interpretation of risk

The communication findings provide additional context for interpreting the awareness results. Environmental-health information can influence how hazards are recognized and discussed, but the present cross-sectional design does not allow the direction of this relationship to be established. Adebayo (15), studying the health-information environment of market women in Offa, Kwara State, demonstrated the importance of information environments within market populations. This is relevant to the present study because market stakeholders may receive environmental-health information through multiple interpersonal, media, and formal channels.

The importance of communication is also supported by broader environmental-health literature. Ramírez et al. (5) argued that environmental-health communication needs to go beyond simply transmitting information and should support the development of environmental-health literacy. Similarly, Adekola et al. (8) highlighted the role of communication and public understanding in the context of environmental degradation and health risks in the Niger Delta. These findings provide a plausible conceptual framework for understanding how respondents may have become familiar with environmental pollutants, but they do not demonstrate that any particular communication channel caused the universal awareness observed in this study.

This distinction is particularly important because the present study found high awareness but heterogeneous practice. Effective communication may contribute to awareness, but behavioral change may require additional conditions, including perceived relevance, practical feasibility, confidence in recommended practices, and enabling circumstances. Chin et al. (18) similarly demonstrated that awareness of air pollution and support for environmental protection did not necessarily translate into equally strong willingness to undertake actions requiring individual effort. Thus, the present findings support an interpretation in which communication is potentially important for awareness and risk understanding, but awareness alone may not be sufficient to ensure consistent protective behavior.

### Integrated interpretation of awareness, risk perception, and practice

Taken together, the findings demonstrate that awareness, risk perception, and food-handling practice represent related but distinct domains. The universal awareness finding indicates that respondents reported recognition of the environmental pollutants assessed. The variation in risk perception indicates that recognition of a hazard did not correspond to a uniform level of perceived risk. The heterogeneity in food-handling practices further indicates that awareness and perception did not translate into a single behavioral pattern.

This interpretation is consistent with the broader knowledge–attitude–practice literature. Desye et al. (20) demonstrated a clear difference between knowledge and practice among street-food vendors in low- and middle-income countries, while Atalay et al. (21) documented persistent variation in food-hygiene practices across sub-Saharan Africa. Nigerian evidence from Itasanmi and Jegede (17) similarly demonstrates that environmental knowledge, attitudes, and behavior may not be perfectly aligned. The present study therefore adds evidence from market stakeholders in Kwara State that reported awareness can coexist with heterogeneous food-handling practices.

The awareness–practice gap may consequently be more informative than the universal-awareness finding considered in isolation. If respondents already recognize environmental pollutants as potential hazards, interventions based solely on repeating general awareness messages may have limited additional value unless they also address the practical translation of knowledge into behavior. This does not mean that awareness-building is unnecessary; rather, it suggests that future interventions could place greater emphasis on practical food-safety skills, context-specific guidance, and conditions that enable safe handling. Such interventions would need to be evaluated empirically because the present study did not test intervention effectiveness.

## Conclusion

This study found that reported awareness of environmental pollutants was universal among the sampled market stakeholders; however, risk perception and food-handling practices varied across markets and respondent characteristics. Importantly, the coexistence of high reported awareness with substantial variation in food-handling practices suggests an important awareness–practice gap, indicating that recognition of environmental and food-safety risks does not necessarily translate into consistent protective practices. Furthermore, the observed associations between respondent characteristics and risk perception should be interpreted as unadjusted associations within the study population rather than as causal determinants.

From a public-health perspective, interventions should therefore move beyond general awareness creation toward practical, context-specific food-safety education, demonstration of appropriate handling practices, and communication strategies that support the translation of risk knowledge into safer behavior. Accordingly, market authorities and relevant public-health agencies should strengthen routine food-safety education and practical hygiene promotion among market stakeholders. In addition, communication programmes should be tailored to the information channels commonly used within market communities.

Nevertheless, the findings should be interpreted in light of several limitations, including the cross-sectional design, self-reported responses, potential social-desirability and recall bias, possible selection bias, the relatively small and geographically restricted sample, and the absence of adjustment for potential confounding. Moreover, the operational categorization of risk-perception and food-handling outcomes may not have been independently validated, and no direct measurements of environmental pollutants or food contamination were undertaken. Consequently, the study cannot establish actual exposure or contamination burden, nor can it demonstrate causal relationships between communication, awareness, risk perception, and food-safety behavior.

In light of these limitations, future studies should use larger and more geographically diverse samples, validated outcome measures, multivariable analytical approaches, and direct environmental and food-contamination measurements. Additionally, longitudinal or intervention-based studies would be valuable for assessing whether targeted risk communication and practical food-safety interventions can produce sustained improvements in protective behavior. Ultimately, the findings suggest that effective food-safety risk communication in market settings should move beyond awareness creation toward interventions that support the practical translation of risk knowledge into consistent protective food-handling behavior.

## Data Availability

The raw data supporting the conclusions of this article will be made available by the authors, without undue reservation.
