# Supplementary material for: Environmental pollutant awareness, food handling practices, and risk communication among market stakeholders in Kwara State, Nigeria: a cross-sectional survey
Source: Front Public Health. 2026 Sep 23;14:1922269. doi: 10.3389/fpubh.2026.1922269 (PMC13642811; doi:10.3389/fpubh.2026.1922269)
Supplement: Supplementary file 1 [file Data_Sheet_1.docx]

**SUPPLEMENTARY MATERIAL**

**Contingency Tables for Environmental Pollutant Risk Perception**

The following supplementary tables present the observed frequencies and row percentages underlying the Pearson chi-square analyses reported in Table 5 of the manuscript. Environmental pollutant risk perception was the dependent categorical variable.

Risk-perception categories comprised Agree, Neutral, and Strongly Agree. The complete dataset contained 120 respondents.

**Supplementary Table S1. Gender by Environmental Pollutant Risk Perception**

| **Gender** | **Agree, n (%)** | **Neutral, n (%)** | **Strongly Agree, n (%)** | **Total, n (%)** |
| --- | --- | --- | --- | --- |
| Female | 5 (6.2) | 0 (0.0) | 75 (93.8) | 80 (66.7) |
| Male | 14 (35.0) | 12 (30.0) | 14 (35.0) | 40 (33.3) |
| Total | 19 (15.8) | 12 (10.0) | 89 (74.2) | 120 (100.0) |

**Note:** Values are observed frequencies with row percentages in parentheses. Percentages within each predictor category are calculated across the three risk-perception categories. The total row shows the overall distribution of environmental pollutant risk perception. These observed frequencies form the contingency table used for the Pearson chi-square test reported in Table 5.

**Supplementary Table S2. Marital Status by Environmental Pollutant Risk Perception**

| **Marital Status** | **Agree, n (%)** | **Neutral, n (%)** | **Strongly Agree, n (%)** | **Total, n (%)** |
| --- | --- | --- | --- | --- |
| Divorced/Widowed | 0 (0.0) | 0 (0.0) | 4 (100.0) | 4 (3.3) |
| Married | 14 (14.9) | 1 (1.1) | 79 (84.0) | 94 (78.3) |
| Single | 5 (22.7) | 11 (50.0) | 6 (27.3) | 22 (18.3) |
| Total | 19 (15.8) | 12 (10.0) | 89 (74.2) | 120 (100.0) |

**Note:** Values are observed frequencies with row percentages in parentheses. Percentages within each predictor category are calculated across the three risk-perception categories. The total row shows the overall distribution of environmental pollutant risk perception. These observed frequencies form the contingency table used for the Pearson chi-square test reported in Table 5.

**Supplementary Table S3. Location by Environmental Pollutant Risk Perception**

| **Location** | **Agree, n (%)** | **Neutral, n (%)** | **Strongly Agree, n (%)** | **Total, n (%)** |
| --- | --- | --- | --- | --- |
| Ganmo | 9 (22.5) | 12 (30.0) | 19 (47.5) | 40 (33.3) |
| Oke-Oyi | 10 (25.0) | 0 (0.0) | 30 (75.0) | 40 (33.3) |
| Oko-Olowo | 0 (0.0) | 0 (0.0) | 40 (100.0) | 40 (33.3) |
| Total | 19 (15.8) | 12 (10.0) | 89 (74.2) | 120 (100.0) |

**Note:** Values are observed frequencies with row percentages in parentheses. Percentages within each predictor category are calculated across the three risk-perception categories. The total row shows the overall distribution of environmental pollutant risk perception. These observed frequencies form the contingency table used for the Pearson chi-square test reported in Table 5.

**Supplementary Table S4. Age Group by Environmental Pollutant Risk Perception**

| **Age Group** | **Agree, n (%)** | **Neutral, n (%)** | **Strongly Agree, n (%)** | **Total, n (%)** |
| --- | --- | --- | --- | --- |
| 18–25 | 6 (31.6) | 7 (36.8) | 6 (31.6) | 19 (15.8) |
| 26–40 | 13 (21.0) | 5 (8.1) | 44 (71.0) | 62 (51.7) |
| 41–60 | 0 (0.0) | 0 (0.0) | 33 (100.0) | 33 (27.5) |
| 60+ | 0 (0.0) | 0 (0.0) | 6 (100.0) | 6 (5.0) |
| Total | 19 (15.8) | 12 (10.0) | 89 (74.2) | 120 (100.0) |

**Note:** Values are observed frequencies with row percentages in parentheses. Percentages within each predictor category are calculated across the three risk-perception categories. The total row shows the overall distribution of environmental pollutant risk perception. These observed frequencies form the contingency table used for the Pearson chi-square test reported in Table 5.

**Supplementary Table S5. Parenting Status by Environmental Pollutant Risk Perception**

| **Parenting Status** | **Agree, n (%)** | **Neutral, n (%)** | **Strongly Agree, n (%)** | **Total, n (%)** |
| --- | --- | --- | --- | --- |
| Kid | 19 (20.9) | 12 (13.2) | 60 (65.9) | 91 (75.8) |
| No kid | 0 (0.0) | 0 (0.0) | 29 (100.0) | 29 (24.2) |
| Total | 19 (15.8) | 12 (10.0) | 89 (74.2) | 120 (100.0) |

**Note:** Values are observed frequencies with row percentages in parentheses. Percentages within each predictor category are calculated across the three risk-perception categories. The total row shows the overall distribution of environmental pollutant risk perception. These observed frequencies form the contingency table used for the Pearson chi-square test reported in Table 5.

**Supplementary Table S6. Income by Environmental Pollutant Risk Perception**

| **Income** | **Agree, n (%)** | **Neutral, n (%)** | **Strongly Agree, n (%)** | **Total, n (%)** |
| --- | --- | --- | --- | --- |
| <₦50,000 | 15 (24.6) | 12 (19.7) | 34 (55.7) | 61 (50.8) |
| >₦250,000 | 0 (0.0) | 0 (0.0) | 16 (100.0) | 16 (13.3) |
| ₦101,000–₦250,000 | 0 (0.0) | 0 (0.0) | 17 (100.0) | 17 (14.2) |
| ₦50,000–₦100,000 | 4 (15.4) | 0 (0.0) | 22 (84.6) | 26 (21.7) |
| Total | 19 (15.8) | 12 (10.0) | 89 (74.2) | 120 (100.0) |

**Note:** Values are observed frequencies with row percentages in parentheses. Percentages within each predictor category are calculated across the three risk-perception categories. The total row shows the overall distribution of environmental pollutant risk perception. These observed frequencies form the contingency table used for the Pearson chi-square test reported in Table 5.

**Supplementary Table S7. Market Role by Environmental Pollutant Risk Perception**

| **Market Role** | **Agree, n (%)** | **Neutral, n (%)** | **Strongly Agree, n (%)** | **Total, n (%)** |
| --- | --- | --- | --- | --- |
| Consumer | 10 (40.0) | 6 (24.0) | 9 (36.0) | 25 (20.8) |
| Official | 0 (0.0) | 0 (0.0) | 6 (100.0) | 6 (5.0) |
| Transporter | 0 (0.0) | 0 (0.0) | 6 (100.0) | 6 (5.0) |
| Vendor | 9 (10.8) | 6 (7.2) | 68 (81.9) | 83 (69.2) |
| Total | 19 (15.8) | 12 (10.0) | 89 (74.2) | 120 (100.0) |

**Note:** Values are observed frequencies with row percentages in parentheses. Percentages within each predictor category are calculated across the three risk-perception categories. The total row shows the overall distribution of environmental pollutant risk perception. These observed frequencies form the contingency table used for the Pearson chi-square test reported in Table 5.

**Supplementary Table S8. Education by Environmental Pollutant Risk Perception**

| **Education** | **Agree, n (%)** | **Neutral, n (%)** | **Strongly Agree, n (%)** | **Total, n (%)** |
| --- | --- | --- | --- | --- |
| Primary | 16 (30.8) | 7 (13.5) | 29 (55.8) | 52 (43.3) |
| Secondary | 3 (6.7) | 5 (11.1) | 37 (82.2) | 45 (37.5) |
| Tertiary | 0 (0.0) | 0 (0.0) | 23 (100.0) | 23 (19.2) |
| Total | 19 (15.8) | 12 (10.0) | 89 (74.2) | 120 (100.0) |

**Note:** Values are observed frequencies with row percentages in parentheses. Percentages within each predictor category are calculated across the three risk-perception categories. The total row shows the overall distribution of environmental pollutant risk perception. These observed frequencies form the contingency table used for the Pearson chi-square test reported in Table 5.

**Food Safety Awareness and Environmental Pollutants in Nigerian Markets: Implications for Public Health Risk Communication**

**Food Safety Awareness and Environmental Pollutants in Nigerian Markets: Implications for Public Health Risk Communication**

You are invited to participate in a vital research study titled "Food Safety Awareness and Environmental Pollutants in Nigerian Markets: Implications for Public Health Risk Communication," which is being carried out by Dr. G. K. Olawepo of the Department of Plant Biology at the University of Ilorin. The objective of this survey is to understand how environmental factors—such as vehicle exhaust, generator soot, and waste disposal—affect the safety of food sold in our local markets. Your feedback will help researchers and health officials develop better ways to communicate health risks and improve food handling practices to protect the Nigerian public from long-term, life-threatening illnesses such as liver damage, kidney disease, and cancer. As a stakeholder in the market ecosystem—whether you are a consumer, vendor, or transporter—your daily experiences provide unique insights that cannot be captured through laboratory tests alone; therefore, your honest responses will contribute directly to a safer food environment for your family and the community. Participation is entirely voluntary, allowing you to skip any question or withdraw at any time, and since no names are required, all responses will be assigned a unique ID and kept strictly confidential to ensure your total anonymity. We kindly ask you to answer the following questions as accurately as possible, noting that there are no "right" or "wrong" answers as we are primarily interested in your personal awareness, concerns, and daily practices. Thank you for your time and for your valuable contribution toward improving public health in Nigeria.

**Dr. G. K. Olawepo**

Lecturer, Department of Plant Biology,

Life Sciences, University of Ilorin

**Section A: Demographic Characteristics**

1. Gender: [ ] Male [ ] Female [ ] Prefer not to say

2. Age Group: [ ] 18–25 [ ] 26–40 [ ] 41–60 [ ] 60+

3. Highest Education Level: [ ] None [ ] Primary [ ] Secondary [ ] Tertiary

4. Primary Role in the Market:

[ ] Consumer (Buyer)

[ ] Vendor/Trader (Seller)

[ ] Transporter (Driver/Loader)

[ ] Market Official/Regulatory Staff

5. Residential Location Type: [ ] Urban (Major city/commercial hub)

[ ] Peri-urban (Suburbs/outskirts)

[ ] Rural (Village/farming community)

6. Marital Status / Parental Status: [ ] Single [ ] Married [ ] Divorced/Widowed[ ]

7. Do you have children under the age of 12? [ ] Yes [ ] No

8. Average Monthly Income (Naira): [ ] Below ₦50,000

[ ] ₦50,000 – ₦100,000

[ ] ₦101,000 – ₦250,000

[ ] Above ₦250,000

**Section B: Awareness of Food Pollutants**

6. Are you aware that food can be contaminated by the environment? [ ] Yes [ ] No

7. Specific Pollutant Identification: Which of the following do you consider to be a "pollutant" found in a market environment? (Check all that apply):

- [ ] Heavy Metals (e.g., Lead from petrol fumes, Cadmium from batteries/waste)
- [ ] Polycyclic Aromatic Hydrocarbons (Soot/smoke from generators or burning refuse)
- [ ] Microplastics (From degraded nylon bags and plastic bottles)
- [ ] Pesticide Residues (Chemicals used to preserve beans or kill weevils)
- [ ] Microbial Pathogens (Bacteria/Parasites from stagnant drainage water)

8. Awareness of Entry Pathways: How do you think these pollutants primarily enter the food sold in the market?

- [ ] Atmospheric Deposition: Dust and exhaust fumes settling on uncovered food.
- [ ] Soil/Water Uptake: Crops absorbing chemicals from contaminated soil or irrigation water before reaching the market.
- [ ] Cross-Contamination: Using dirty water to wash vegetables or placing food on chemically treated surfaces.
- [ ] Bioaccumulation: Animals (like fish or cattle) eating pollutants, which then stay in their meat/milk.

9. Visual vs. Invisible Detection: Do you believe a food item can look "clean" and "fresh" but still be dangerously contaminated by chemicals or heavy metals?

- [ ] Yes [ ] No [ ] Not Sure

10. Persistence Awareness: Which of these do you believe can effectively remove chemical pollutants (like Lead or Pesticides) from food? (Check all that apply):

- [ ] Washing with water only
- [ ] Washing with salt or vinegar
- [ ] Parboiling/Cooking at high heat
- [ ] Peeling the outer skin
- [ ] None of the above (Correct scientific view for certain heavy metals)

11. Awareness of Local Sources: Are you aware of any specific activities near this market that could contribute to food pollution?

- [ ] Nearby Mechanic workshops (Oil/Lead spills)
- [ ] Open refuse burning (Soot/Dioxins)
- [ ] Heavy truck/bus traffic (Carbon monoxide/Exhaust)
- [ ] Use of "Sniper" or other chemicals for preservation by traders
- [ ] Proximity to industrial discharge or dirty gutters

**Section C: Risk Perception (Perceived Severity & Vulnerability)**

*Please rate your level of agreement with the following statements:*

*(Scale: 1 = Strongly Disagree, 2 = Disagree, 3 = Neutral, 4 = Agree, 5 = Strongly Agree)*

1. Perceived Severity of Health Damage

- "I believe environmental pollutants in food (like heavy metals from smoke) can cause permanent, irreversible damage to the liver or kidneys." [1—2—3—4—5]
- "I consider the long-term health risks of food pollutants to be more life-threatening than immediate illnesses like typhoid or malaria." [1—2—3—4—5]
- "I believe that consuming even small amounts of these pollutants over many years can eventually lead to terminal diseases like cancer." [1—2—3—4—5]

2. Perceived Susceptibility & Vulnerability

- "I believe that I am personally at risk of health problems because of where I buy or sell food in this market." [1—2—3—4—5]
- "I believe that children and the elderly are at a much higher risk of permanent brain or growth damage from these pollutants than healthy adults." [1—2—3—4—5]
- "I am concerned that food grown in Nigeria may already be contaminated by pollutants before it even reaches the market." [1—2—3—4—5]

3. Socio-Economic Risk Impact

- "The financial cost of treating a chronic illness caused by food pollutants would be impossible for my family to afford." [1—2—3—4—5]

**Section D: Food Safety and Handling Practices**

1. Point-of-Sale Protection (Market Display) *How are the food items you buy/sell typically protected from the environment?*

- [ ] Displayed openly on tables/ground
- [ ] Partially covered with nylon or cloth
- [ ] Fully enclosed in glass/plastic containers or display cases
- [ ] Elevated on high platforms away from the floor/gutter

2. Spatial Risk Management (Location Selection) *When selecting a stall or displaying goods, how often do you avoid areas near the following?* *(Scale: 1 = Never Avoid, 2 = Rarely, 3 = Sometimes, 4 = Usually, 5 = Always)*

- Heavy traffic, bus stops, or idling vehicles: [1—2—3—4—5]
- Refuse dump sites or overflowing garbage: [1—2—3—4—5]

3. Safety Prioritization *When you think of "safe food," which of the following is your primary focus?*

- [ ] Biological Safety: Removing germs, bacteria, and dirt that cause immediate sickness (e.g., diarrhea).
- [ ] Chemical Safety: Removing invisible pollutants, heavy metals, and pesticides that cause long-term damage (e.g., organ failure).
- [ ] Both equally.

4. Decontamination and Preparation (At Home/Stall) *Which methods do you use to remove environmental pollutants (dust, soot, chemicals) before consumption? (Check all that apply):*

- [ ] Washing with plain water only
- [ ] Washing with additives (Salt, Vinegar, Lemon, or Potash)
- [ ] Parboiling and discarding the initial water
- [ ] Peeling or scraping the outer skin of fruits/tubers
- [ ] Using a brush to scrub the surface of the food
- [ ] None (I believe the pollutants cannot be removed)

5. Transportation and Logistics *How is the food typically moved from the farm/wholesale point to the market or home?*

- [ ] Carried in open-back trucks/vans (Exposed to road dust and exhaust)
- [ ] Carried in closed vehicles or sealed bags
- [ ] Transported alongside non-food items (e.g., fuel cans, fertilizers, or pesticides)
- [ ] Transported in contact with the vehicle floor/dirty surfaces

**Section E: Information Sources & Risk Communication**

1. Primary Information Channels *Where have you heard or read about food safety and environmental pollutants in the last 6 months? (Check all that apply):*

- [ ] Traditional Media (Radio, TV, Newspapers)
- [ ] Digital Media (WhatsApp, Facebook, TikTok)
- [ ] Religious Institutions (Church/Mosque)
- [ ] Market/Trade Union Meetings
- [ ] Community Town Criers or Megaphones
- [ ] Family, Friends, and Neighbors
- [ ] Government Health Officers/Sanitarians (Wole-Wole)

2. Trust Hierarchy *Who do you trust most to give you truthful information about food pollutants? (Rank top 3, where 1 is Most Trusted):*

- [ ] Government Agencies (e.g., NAFDAC, Ministry of Health) [ ]
- [ ] Scientists/Researchers [ ]
- [ ] Market Union Leaders/Iyaloja/Babaloja [ ]
- [ ] Religious Leaders [ ]
- [ ] Social Media Influencers [ ]
- [ ] Health Workers (Doctors/Nurses) [ ]

3. Preferred Communication Format *Which method would be most effective in changing your food handling behavior?*

- [ ] Audio messages in local languages (Radio/WhatsApp voice notes)
- [ ] Visual posters and infographics (Pictures showing "Bad" vs "Good")
- [ ] One-on-one training or community workshops
- [ ] Short video clips (Drama-style)
- [ ] Formal text-based flyers (English/Pidgin)

4. Communication Frequency & Accessibility

- How often do you encounter news or warnings about environmental pollutants (like heavy metals or generator soot) in the market?
  - [ ] Never [ ] Rarely [ ] Monthly [ ] Weekly

5. Language & Cultural Context *Which language would make you feel the information is "for you" and should be taken seriously?*

- [ ] English
- [ ] Pidgin English
- [ ] Local Language (Yoruba/Igbo/Hausa)
- [ ] A mix (e.g., English explained in local dialect)

6. Perceived Effectiveness of Current Communication

- "I feel that currently, the government does enough to inform people about the dangers of pollutants in the market."
  - [1—2—3—4—5] *(Strongly Disagree to Strongly Agree)*
- "I would be more likely to follow safety advice if it were delivered by my Market Union Leader rather than a government official."
  - [1—2—3—4—5]
